# Neutrophil Functions in Periodontal Homeostasis

**DOI:** 10.1155/2016/1396106

**Published:** 2016-02-25

**Authors:** Ricarda Cortés-Vieyra, Carlos Rosales, Eileen Uribe-Querol

**Affiliations:** ^1^Departamento de Inmunología, Instituto de Investigaciones Biomédicas, Universidad Nacional Autónoma de México, 04510 Ciudad de México, DF, Mexico; ^2^División de Estudios de Posgrado e Investigación, Facultad de Odontología, Universidad Nacional Autónoma de México, 04510 Ciudad de México, DF, Mexico

## Abstract

Oral tissues are constantly exposed to damage from the mechanical effort of eating and to microorganisms, mostly bacteria. In healthy gingiva tissue remodeling and a balance between bacteria and innate immune cells are maintained. However, excess of bacteria biofilm (plaque) creates an inflammation state that recruits more immune cells, mainly neutrophils to the gingiva. Neutrophils create a barrier for bacteria to reach inside tissues. When neutrophils are insufficient, bacteria thrive causing more inflammation that has been associated with systemic effects on other conditions such as atherosclerosis, diabetes, and cancer. But paradoxically when neutrophils persist, they can also promote a chronic inflammatory state that leads to periodontitis, a condition that leads to damage of the bone-supporting tissues. In periodontitis, bone loss is a serious complication. How a neutrophil balance is needed for maintaining healthy oral tissues is the focus of this review. We present recent evidence on how alterations in neutrophil number and function can lead to inflammatory bone loss, and how some oral bacteria signal neutrophils to block their antimicrobial functions and promote an inflammatory state. Also, based on this new information, novel therapeutic approaches are discussed.

## 1. Introduction

Neutrophils are the most abundant leukocytes in blood and are considered to be the first line of defense during inflammation and infections [1]. Invading microorganisms evoke an inflammatory response that recruits neutrophils from the circulation into the tissues. There, neutrophils destroy the microorganism by a series of mechanisms, mainly phagocytosis, release of antimicrobial substances, and the formation of neutrophil extracellular traps (NETs) [2]. Activated neutrophils also release proteinases into the surrounding tissue, causing damage to the host [3], and can live for much longer than previously thought. It is estimated that neutrophil half-life is days instead of hours [4]. In addition, neutrophils are capable of producing many cytokines and chemokines, which can influence the inflammatory response, as well as the immune response [5].

Periodontitis is a chronic inflammatory disease that destroys the tooth-supporting tissues or periodontium. Tissues involved are the gingiva, the periodontal ligament, and the alveolar bone. It is estimated that, in the USA alone, 46% of the population over 30 years of age present periodontitis, and 8.9% have severe periodontitis [6]. Periodontium damage leads to tooth loss, and in severe cases it can also affect systemic health by increasing a person's risk for atherosclerosis, rheumatoid arthritis, diabetes, and even cancer [6].

Periodontal diseases stimulate ecological imbalances in the mouth that promote the growth of selected commensal bacteria that induce host inflammatory pathways [7–9]. A clear correlation between periodontal disease and atherosclerosis has been established in clinical observations and animal models [10–12]. In particular polymicrobial infection with* Porphyromonas gingivalis*,* Treponema denticola*,* Tannerella forsythia*, and* Fusobacterium nucleatum* has been shown to promote progression of atherosclerosis [11–13]. In hyperlipidemic ApoE^(−/−)^ mice, a systemic infection with* P. gingivalis* genomic DNA was indicated by the detection of bacterial genomic DNA in the aorta, liver, and spleen tissues of infected mice [10, 12]. Bacterial products could activate innate immune cells through the NLRP3 inflammasome [10] to produce elevated levels of bacterial specific IgG antibodies [12]. Consequently, the host immune response to bacterial products, for example, the heat-shock protein GroEL of* F. nucleatum*, may be a mechanism involved in atherosclerosis [13]. Similarly, many studies have confirmed an association between periodontitis and diabetes. Higher plaque levels and higher incidence of chronic gingivitis are both found in adults and in children with diabetes [14, 15]. Susceptibility to periodontitis augments threefold in people with hyperglycemia, which aggravates tissue destruction and periodontitis [16]. At the same time, there is evidence indicating a bidirectional relationship between diabetes and periodontal disease; not only does diabetes increase the risk for periodontitis, but also periodontitis is associated with compromised glycemic control [16]. Furthermore, periodontal treatment showed a beneficial effect on metabolic control of type 2 diabetic patients [15]. The cellular and molecular mechanisms for these interactions are complex and remain unknown for the most part. Nonetheless, an exacerbated inflammatory state seems to be a common denominator [16, 17]. The same is true for the influence of periodontal disease and cancer [18].

**Figure 1:**
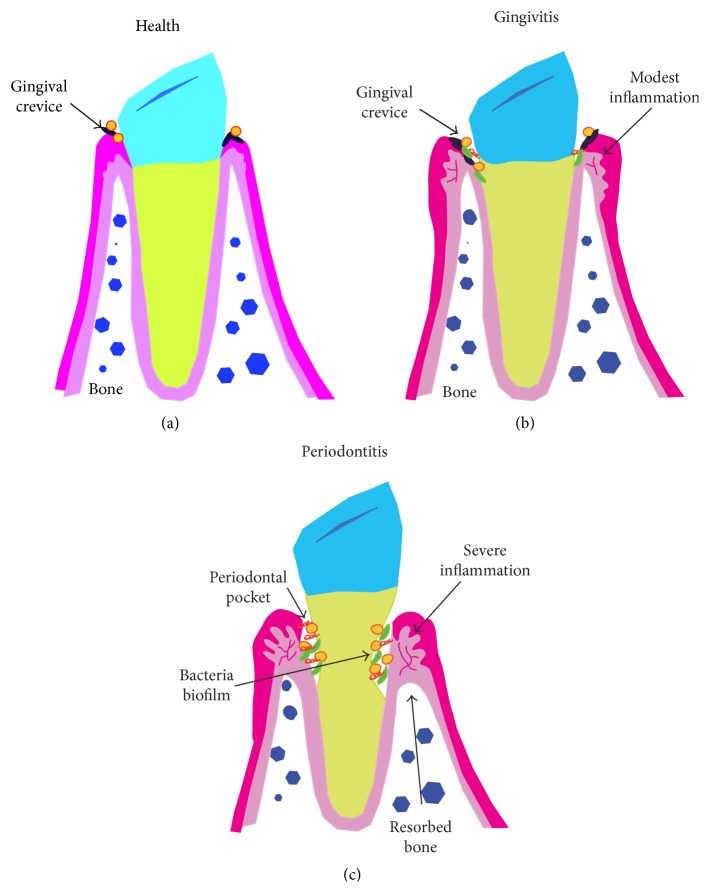
*The evolution from a healthy periodontium to periodontitis*. (a) Healthy tooth with no inflammation and a small gingival crevice (space between the tooth surface and the free gingiva). (b) A change in oral microbiota leads to increased bacteria biofilm deposition in the gingival crevice and produces moderate inflammation (gingivitis). (c) In severe cases, a chronic inflammatory state known as periodontitis is established, resulting in the formation of periodontal pockets (pathologically deepened gingival crevices) with extensive tissue destruction including bone loss.

The evolution from a healthy periodontium (Figure 1(a)) to periodontitis is associated with a change in oral microbiota from symbiotic bacteria to dysbiotic anaerobic microorganisms, which have adapted to thrive in an inflammatory environment. Bacteria, such as* Porphyromonas gingivalis*, induce changes in the normal microbiota of the gingival crevicular fluid, leading to increased biofilm deposition in the gingival crevice (space between the tooth surface and the free gingiva) and moderate inflammation (gingivitis) (Figure 1(b)). In severe cases, a chronic inflammatory state is established, resulting in the formation of periodontal pockets (pathologically deepened gingival crevices) with extensive tissue destruction including bone loss (Figure 1(c)).

An alteration in the balance, dysbiosis, of bacteria oral microbiota is thought to be the initial trigger for periodontitis [19]. The accumulation of bacteria biofilm leads to an increase in the inflammatory infiltrate, composed mainly by neutrophils into oral tissues. There, neutrophils form a barrier that prevents bacteria from invading deeper tissues. Thus, lack of neutrophils leads to severe periodontitis [20, 21]. Unfortunately, excess of neutrophils seems also to be detrimental by maintaining an inflammation state that leads to tissue destruction. Some oral bacteria have developed strategies to disrupt neutrophils function in order to boost the inflammation state and to promote their persistence in periodontitis.

It is the purpose of this review to highlight how a neutrophil balance is needed for maintaining healthy oral tissues and to describe recent studies that provide some light on the mechanisms for neutrophil recruitment to gingival tissues, for how both lack of and excess of neutrophils lead to inflammation-mediated bone loss, and for oral bacteria destabilization of neutrophil function. In addition, we discuss some of the novel therapeutic approaches to treat chronic inflammation in oral tissues.

## 2. Neutrophils Homeostasis

Because neutrophils are the principal leukocyte (>95%) recruited to the gingival crevice in response to bacteria biofilm deposited on teeth [20, 22], and their absence or excess leads to periodontal tissue damage, it is evident that neutrophil numbers and distribution play a fundamental role in maintaining oral health. Neutrophil homeostasis is important to prevent collateral damage to the host by the potent antimicrobial and proinflammatory effects of these cells. Neutrophil homeostasis involves several mechanisms at the level of production, trafficking, and clearance [23].

### 2.1. Neutrophil Production

**Figure 2:**
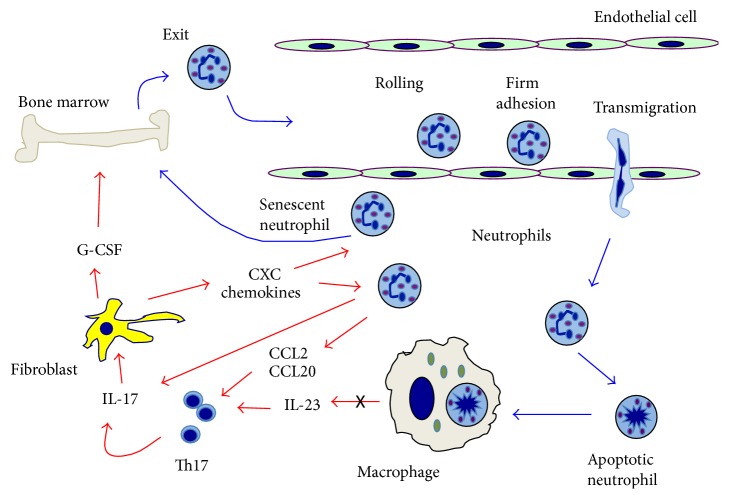
*Neutrophil trafficking*. A neutrophil rheostat (neutrostat) that maintains steady-state neutrophil levels involves the axis interleukin- (IL-) 23/IL-17/granulocyte colony-stimulating factor (G-CSF). Granulopoiesis takes place in the bone marrow where proliferation of granulocytic precursors is induced by G-CSF. The exit of mature neutrophils from the bone marrow into the blood circulation is also regulated by G-CSF. Circulating neutrophils roll on endothelial cells at sites of infection of inflammation. Upon firm adhesion mediated by integrins, neutrophils can transmigrate into the tissues. There, neutrophils perform their antimicrobial functions and then die by apoptosis. Apoptotic neutrophils are phagocytosed by macrophages. This initiates an anti-inflammatory signal that reduces IL-23 production from macrophages (black cross). IL-23 can activate Th17 lymphocytes to produce IL-17, which promotes granulopoiesis and neutrophil release by upregulation of G-CSF in fibroblasts. An interesting positive loop for neutrophil recruitment is found at chronic inflammation sites, where neutrophils can attract Th17 cells via CCL2 and CCL20 chemokines. In turn, Th17 cells recruit more neutrophils via CXC chemokines by inducing neutrophils to produce IL-17. Senescent neutrophils return to the bone marrow for clearance after they increase expression of CXCR4. Red arrows represent release of cytokines and chemokines from cells, and effect of these mediators on cells. Blue arrows represent movement of cells.

Neutrophils are produced in large numbers in the bone marrow and released daily into the circulation [24]. Granulopoiesis and neutrophil release are regulated primarily by the granulocyte colony-stimulating factor (G-CSF). G-CSF promotes proliferation of granulocytic precursors in the bone marrow and exit of mature neutrophils [23]. There is a large pool of mature neutrophils in the bone marrow that is retained there by the interaction of the chemokine CXCL12 (stromal-derived factor-1/SDF-1) produced by the bone marrow stromal cells, with the CXC chemokine receptor 4 (CXCR4) on neutrophils. G-CSF induces neutrophil exit from the bone marrow by interfering with the CXCR4-CXCL12 interaction [25]. In addition, interleukin-17 (IL-17) promotes granulopoiesis and neutrophil release by upregulation of G-CSF [23] (Figure 2). An interesting positive loop for neutrophil recruitment is found at chronic inflammation sites, where neutrophils can attract IL-17-producing CD4^+^ T lymphocytes (Th17 cells) [26]. In addition, neutrophil themselves are an important source of IL-17 [27]. Therefore, the production of IL-17 by neutrophils is also a part of this positive loop for neutrophil recruitment (Figure 2). Neutrophils release CCL2 and CCL20 chemokines, which are ligands for the CCR2 and CCR6 chemokine receptors on Th17 cells, and thus they are recruited to sites of neutrophil accumulation. In turn, Th17 cells recruit more neutrophils [28, 29] (Figure 2).

### 2.2. Neutrophil Trafficking

Once neutrophils are in the circulation, they can be quickly mobilized to sites of infection or inflammation through a highly controlled process known as the leukocyte adhesion cascade [30, 31]. First, neutrophils roll on activated endothelial cells, which express adhesion receptors such as E-, and P-selectins. This rolling depends on transient interactions of selectins with glycoprotein ligands on neutrophils. Then, the neutrophil gets activated by chemokines, which induce a high affinity state in another group of adhesion receptors, the integrins. Interaction of both selectins and integrins with their corresponding ligands leads to slow rolling and then to firm adhesion bringing the neutrophil to a full stop. Next the neutrophil crawls on the endothelium and finally transmigrates into peripheral tissues. Transmigration is regulated mainly by *β*2 integrins. These are heterodimeric receptors formed by a unique *α* (CD11) and a common *β* (CD18) subunit that interact with adhesion ligands such as intercellular adhesion molecule-1 (ICAM-1) and ICAM-2 on endothelial cells. The integrin LFA-1 (CD11a/CD18) is involved in firm adhesion, while the integrin Mac-1 (CD11b/CD18) participates in crawling and transmigration [32]. This leukocyte adhesion cascade can be regulated by tissue-derived cytokines, which control the expression of endothelial adhesion molecules, and by tissue-derived chemokines, which induce integrin conformational changes that result in their high affinity state [33]. Once neutrophils are in the peripheral tissues, they follow gradients of chemoattractants in inflamed or infected sites. Potent chemoattractants for neutrophils are some bacterial components such as formyl-methionyl-leucyl-phenylalanine (fMLF), and activated complement components such as the anaphylatoxin C5a [33].

All molecules described above promote the various steps of the leukocyte adhesion cascade in order to achieve neutrophil extravasation. In contrast, negative regulators of this process were unknown until very recently. Del-1, pentraxin 3, and growth-differentiation factor 15 are newly described endogenous inhibitors for leukocyte recruitment [32]. Del-1 (developmental endothelial locus-1), a 52-kDa endothelial-cell secreted glycoprotein, is the first negative regulator of *β*2 integrin-dependent neutrophil recruitment [34]. Del-1 blocks the interaction between LFA-1 or Mac-1 integrins to ICAM-1 on endothelial cells [34], thus preventing firm adhesion and consequently transendothelial migration [27].

### 2.3. Neutrophil Clearance

Neutrophils in tissues undergo apoptosis and then are cleared locally by resident phagocytes, such as macrophages and dendritic cells. Senescent neutrophils in blood return to the bone marrow for clearance after they increase expression of CXCR4 [35]. The clearance of apoptotic neutrophils is more than just elimination of old cells, since it is important for the control of neutrophil production in the bone marrow [36]. Phagocytosis of apoptotic neutrophil triggers an anti-inflammatory response characterized by a reduction in IL-23 production by macrophages. IL-23 is a major cytokine for inducing IL-17 production by many cells of the immune system [36] (Figure 2). Thus, the reduced IL-17 levels lead to less G-CSF production and in consequence less neutrophil production [36]. This control loop has been described as a “neutrostat” (neutrophil rheostat) that maintains steady-state neutrophil levels (Figure 2).

## 3. Neutrophils in Periodontitis

As mentioned before, neutrophils are the main leukocytes recruited to the gingival crevice. Neutrophils exit the gingiva blood vessels and travel through the gingival junctional epithelium until they reach the crevice [22]. At the gingival crevice, neutrophils create a barrier against the growing bacteria biofilm. This neutrophil wall is thought to prevent bacteria form invading the underlying tissues [37]. Migration of neutrophils in the gingiva is controlled by gradients of chemokines and adhesion molecules. Gradients of CXCL8 (IL-8), ICAM-1, and E-selectin have been observed along the pathway neutrophils travel towards the gingival crevice [38]. The neutrophil recruitment to the periodontium was found in mice, to be dependent primarily on the CXC chemokine receptor 2 (CXCR2), which binds to neutrophil-specific chemoattractants such as CXCL1 and CXCL2 (the murine analogs of human IL-8) [39]. This result agrees with the important role of CXC chemoattractants described for the initial phase of directed extravasation of neutrophils [33]. Contrary to what was expected, neutrophil recruitment to periodontium is independent of commensal bacteria, since neutrophils were found in the gingiva of germ-free mice [39]. Thus, it is possible that neutrophil recruitment to the gingival tissues is not only to control bacterial infections, but also to regulate neutrophil homeostasis [20].

The presence of neutrophils is required for preserving oral health. This is made evident by the fact that individuals with defects in production and distribution of neutrophils develop severe forms of periodontitis [38]. Some of these defects are rare and congenital and include the Chediak-Higashi syndrome, Papillon-Lefèvre syndrome, neutropenias, and leukocyte adhesion deficiency (LAD) [40]. In the Chediak-Higashi syndrome and the Papillon-Lefèvre syndrome, neutrophils have defective chemotaxis and therefore they are not recruited properly to sites of infection. Patients with these syndromes develop severe and rapid periodontal bone loss at very young age [40, 41]. Particularly, the Papillon-Lefèvre syndrome results from mutations that inactivate the cysteine protease cathepsin C, which processes a variety of serine proteases considered essential for antimicrobial defense. Thus, neutrophils from these patients released lower levels of LL-37, an antimicrobial peptide with activity against* Actinobacillus actinomycetemcomitans* [42]. Also, LL-37 was totally absent in gingival crevicular fluid from Papillon-Lefèvre syndrome patients despite the large amounts of its precursor, hCAP18 [43]. The lack of LL-37 correlated with a high prevalence of* Aggregatibacter actinomycetemcomitans* infection [43]. Together these reports suggest a direct link between neutrophil functioning and difficulty in controlling periodontitis-associated infections. In addition, neutrophils from another Papillon-Lefèvre syndrome patient did not express the major neutrophil serine proteases, elastase, cathepsin G, and proteinase 3 and were unable to form NETs in response to ROS and also to form LL-37 [44]. However, this patient did not show evidence of an increased tendency toward bacterial infections [44]. Therefore, it seems that serine proteases are needed not only for production of antimicrobial peptides, but also for maintenance of inflammatory homeostasis within gingival crevices [45].

Same conditions of periodontitis and periodontal bone loss are observed in CXCR2-deficient mice, which cannot recruit neutrophils to oral tissues and develop severe bone loss early in life [39]. Neutropenia, a persistent reduction of neutrophil numbers in circulation (<1500 cell/*μ*L), is often associated with susceptibility to infections. In many neutropenic conditions periodontal disease is also frequent and severe, involving the primary and the permanent dentition [46]. LAD is a group of inherited disorders, in which neutrophils cannot leave the circulation and migrate to sites of inflammation or infection [47]. Neutrophils of LAD patients have defective expression and function of *β*2 integrins or other adhesion molecules. Therefore these cells cannot adhere to the vascular endothelium and transmigrate out of the circulation. Three types of LAD are found: LAD-I does not express *β*2 integrins, LAD-II has defective glycosylation of selectin ligands, and LAD-III cannot activate integrins properly [41]. LAD-I is the most common type and patients with this condition present very few neutrophils in peripheral tissues despite of a strong neutrophilia (large numbers of neutrophils in circulation). Patients suffer from recurrent infections and also develop severe periodontitis early in life [41].

The importance of neutrophils for maintaining healthy oral tissues is underscored by the fact that severe forms of periodontitis develop in all congenital conditions with deficiencies in neutrophil numbers and function. However, the presence of neutrophils is not always protective. In fact, the numbers of neutrophils in inflamed periodontal tissues correlate with the severity of the lesions [48], and the tissue destruction seems to be collateral damage of hyperactive neutrophils [37, 49, 50]. In addition, severe periodontitis is associated with chronic inflammation, and neutrophils have been shown to be potent modulators of inflammation and the immune response [51, 52]. Thus it seems that neutrophils can contribute to periodontal tissue damage not only by their enhanced activity, but also by deregulating the gingival environment. An example of this has been mentioned earlier. Neutrophils can recruit Th17 cells and these cells in turn can recruit more neutrophils [28, 29] (Figure 2). This will generate a vicious cycle where the inflammation state persists indefinitely. Supporting this view is the fact that although in the gingival crevice and periodontal pockets, leukocyte infiltrate is almost exclusively made of neutrophils, in the underlying connective tissue of periodontal lesions about 40% of the infiltrating leukocytes are lymphocytes [22].

## 4. Inflammation in Periodontitis

Neutrophils are essential for maintaining oral tissues healthy. In the case of neutrophils deficiencies, severe periodontitis appears with a concomitant inflammation state. On the other hand, in the case of excess numbers of neutrophils also a chronic inflammatory state is present. Thus, inflammation is an important element in periodontitis that is deregulated when neutrophil homeostasis is altered. The mechanisms for creating an inflammatory environment in each case are beginning to be resolved and are described next.

### 4.1. Inflammation in the Absence of Neutrophils

As mentioned before, LAD-I patients present frequent infections and develop early severe periodontitis, despite neutrophilia. This periodontitis has traditionally been explained by the lack of neutrophil control on bacteria infections [53, 54]. However, this type of periodontitis does not usually respond to treatment with antibiotics or mechanical removal of bacteria biofilm [55], suggesting that other mechanisms are at work. Recently it was shown that the driving force for this type of periodontitis involves the production of IL-23 and IL-17 [21]. In LAD-1 patients or in LFA-1 deficient mice, T cells were identified as the main producers of IL-17 [21]. In mice these lymphocytes were *γδ* T cells and in humans they probably are Th17 cells [56]. IL-17 not only stimulates fibroblasts to produce G-CSF but also promotes inflammation and stimulates osteoclasts, leading to bone loss both in humans and in animal models of arthritis [57, 58]. These findings are in agreement with the neutrostat mechanism discussed above. When apoptotic neutrophils are phagocytosed by macrophages, anti-inflammatory signals are produced that lead to less IL-23 production, which is a strong inducer for IL-17 production. IL-17 in turn induces G-CSF production (Figure 2). In LAD-1 individuals, the IL-23/IL-17/G-CSF axis is deregulated resulting in increased granulopoiesis and neutrophilia, and also in IL-17-mediated bone loss [21]. In addition, the inflammatory state creates a nutritionally favorable environment for bacterial growth from tissue-breakdown products [59]. Interestingly, although bacteria can grow without neutrophil surveillance in the periodontal pockets, they do not show uncontrolled invasion of the underlying connective tissue. Instead, bacterial products translocate into the underlying tissues where they promote deregulation of the IL-23/IL-17 axis [60, 61]. Similar findings of altered IL-23 and IL-17 responses were also reported in the chemokine receptor CXCR2-deficient mice, which do not recruit neutrophils to periodontal tissues and have susceptibility to periodontitis [39]. Therefore, in LAD-1 periodontitis tissue destruction is a consequence of the absence of neutrophils that leads to an alteration of the neutrostat mechanism, inducing inflammation and bone loss.

### 4.2. Inflammation in the Excess of Neutrophils

Neutrophils can also be found in large numbers in inflamed periodontal tissues, and their presence correlates with severity of the lesions [48] and the tissue destruction seems to be collateral damage of hyperactive neutrophils [37, 49, 50]. As mentioned above, neutrophil recruitment is at least in part regulated by Del-1 and LFA-1 interactions. Del-1 blocks LFA-1 binding to its ligand ICAM-1 and prevents neutrophil extravasation [34]. Accordingly, Del-1-deficient mice present excess of neutrophil infiltration and develop spontaneous periodontitis [27]. This inflammation was also shown to be associated with elevated IL-17 levels, and antibodies against IL-17 prevented inflammation and bone loss placing IL-17 as responsible for the tissue damage. This was confirmed with mice double deficient in Del-1 and IL-17R. These animals were protected from periodontal disease [27]. The source of IL-17 is mainly T lymphocytes, which can be recruited by neutrophils (Figure 2), but also neutrophil themselves are an important source of IL-17 [27], as shown in a subpopulation of neutrophils expressing the transcription factor retinoic acid-related orphan receptor *γδ* and producing IL-17 [62]. It was also found that Del-1 and IL-17 regulate each other in the periodontium. While Del-1 blocks integrin-dependent neutrophil migration into tissues and IL-17 production, IL-17 inhibits Del-1 expression on endothelial cells, promoting neutrophil infiltration [27]. Thus, IL-17 can promote a signaling loop that would lead to persistent neutrophil recruitment and chronic inflammation. High levels of IL-17 could be responsible for the bone loss in chronic periodontitis, by stimulating osteoblast expression of RANKL, an important osteoclastogenesis factor [63]. In addition, neutrophils activated through TLR can also express RANKL and induce osteoclastogenesis [64]. Very recently another aspect of osteoclast regulation was revealed. Del-1 is expressed on human and mouse osteoclasts and regulates their differentiation and bone resorptive functions. Del-1 inhibited expression of NFATc1, a key transcription factor for osteoclastogenesis, and when administered locally Del-1 blocked inflammatory periodontal bone loss in nonhuman primates [65].

Together these reports confirm that absence of neutrophils as in LDA-1 patients and excess on neutrophils as in Del-1 deficient animals are two sides of altered neutrophil homeostasis that lead by different mechanisms to IL-17-dependent inflammation and bone loss. The fact that Del-1 can prevent this problem in a nonhuman primate model of periodontitis suggests that new therapeutic approaches may be coming available to treat periodontitis.

## 5. Bacterial Control of Neutrophil Function

The studies presented above clearly have shown that in patients with severe forms of periodontitis, more neutrophils with a prosurvival phenotype are present [66]. These cells should control and even eliminate bacteria growth, but instead they promote an inflammation state. The persisting bacteria biofilm functions as a constant stimulus for more neutrophil recruitment. How are these oral bacteria able to remain even among large numbers of neutrophil phagocytes? Some of this bacteria display a survival mechanism that takes control of neutrophil functions, preventing microbial killing and promoting inflammation [67]. This subversion of neutrophils has been described in great detail molecularly, and it is worth reviewing here.

**Figure 3:**
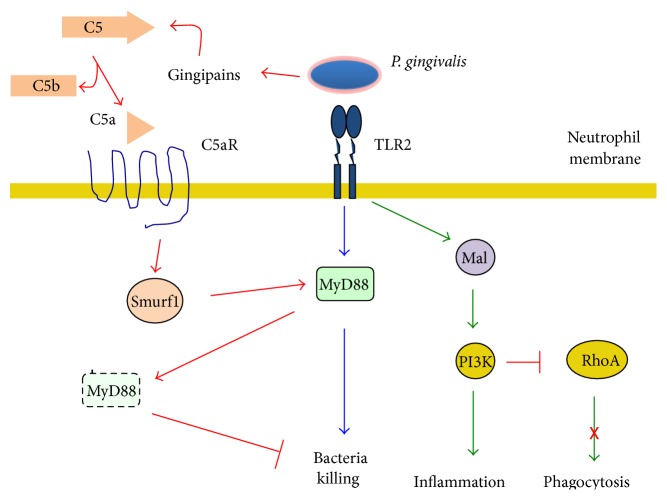
*P. gingivalis can manipulate both complement and TLR signaling to induce bacterial persistence*. The bacterium* P. gingivalis* is recognized by the TLR2 on neutrophils and activates the adaptor protein MyD88 for bacterial elimination (blue arrows). However,* P. gingivalis* produce gingipains, enzymes that function as complement C5 convertases. C5a complement fragment binds to its receptor (C5aR), which induces the E3 ligase Smurf1 to ubiquitinate MyD88 and mark if for proteosomal degradation (red arrows), effectively inhibiting bacteria killing. Interestingly, binding of* P. gingivalis* to the TLR2 also induces inflammation through a signaling pathway involving the adaptor molecule Mal and the enzyme phosphatidylinositol 3-kinase (PI-3K) (green arrows). Also, the same pathway can prevent actin polymerization through inhibition of the small GTPase RhoA and block phagocytosis.

*P. gingivalis*, a key bacterium in periodontitis, can manipulate both complement and TLR signaling to induce bacterial persistence [68]. TLR2 has been shown to be important for bacterial elimination through activation of the adaptor protein MyD88 [69] (Figure 3). But this pathway can be inhibited by activation of a cross talk between TLR2 and C5aR in neutrophils that leads to proteosomal degradation of MyD88 [68] (Figure 3).* P. gingivalis* produce gingipains, enzymes that function as complement C5 convertases, that can produce large amounts of C5a independently of complement activation [70, 71]. C5a binds then to the C5a receptor, which induces the E3 ligase to ubiquitinate MyD88 and mark it for degradation (Figure 3), effectively inhibiting bacteria killing. Interestingly,* P. gingivalis* also induces inflammation from the neutrophil by the same strategy. Binding to the TLR2 an alternative signaling pathway involving the adaptor molecule Mal and the enzyme phosphatidylinositol 3-kinase (PI-3K) a strong inflammatory response is induced (Figure 3). As if this was not enough, the same pathway can prevent actin polymerization through the small GTPase RhoA and block phagocytosis. By this mechanism* P. gingivalis* in addition protects other bystander bacteria from neutrophil microbial mechanisms [68] (Figure 3). As a result,* P. gingivalis* controls neutrophil function by two mechanisms that together ensure bacterial survival and continuation of inflammation [61].

## 6. Potential Therapeutic Approaches

From the literature discussed, it is clear that both lack of neutrophils as well as excess of neutrophils to the periodontium can lead to periodontal inflammation. Because both situations involve IL-17-mediated inflammation and bone loss, it is conceivable that IL-17 or IL-17R inhibitors may be promising for the treatment of human periodontitis [72].

Also in chronic periodontitis, the neutrophil subversion pathways activated by periodontal bacteria are promising targets for treatment of this disease. In fact, promising results in preclinical models of periodontitis have been reported with complement inhibitors [68, 73, 74].

Del-1 is another promising candidate molecule to be used therapeutically to prevent neutrophil recruitment and bone loss associated with periodontal inflammation [27]. In fact, very recently it was reported that locally administered human Del-1 prevented inflammatory bone loss in a model of periodontitis with nonhuman primates [65].

Thus, new therapeutic candidates for treating periodontitis are now in preclinical studies.

## 7. Conclusion

Oral tissues require a constant surveillance by neutrophils in order to remain healthy. When alterations in the neutrophil homeostasis develop various forms of periodontitis appear. Both defects in recruitment and defects in proper function of neutrophils lead to periodontitis, although through different mechanisms. Neutrophils can also then affect oral tissues when they are altered by systemic conditions, such as diabetes mellitus [75]. From the mechanistic studies presented above, new potential therapeutic approaches have been identified. They promise a relief from periodontitis and perhaps other inflammatory disorders.

## References

[1] Borregaard N. (2010). Neutrophils, from marrow to microbes. *Immunity*.

[2] Nauseef W. M. (2007). How human neutrophils kill and degrade microbes: an integrated view. *Immunological Reviews*.

[3] Mayadas T. N., Cullere X., Lowell C. A. (2014). The multifaceted functions of neutrophils. *Annual Review of Pathology: Mechanisms of Disease*.

[4] Tak T., Tesselaar K., Pillay J., Borghans J. A. M., Koenderman L. (2013). What's your age again? Determination of human neutrophil half-lives revisited. *Journal of Leukocyte Biology*.

[5] Mocsai A. (2013). Diverse novel functions of neutrophils in immunity, inflammation, and beyond. *The Journal of Experimental Medicine*.

[6] Eke P. I., Dye B. A., Wei L. (2015). Update on prevalence of periodontitis in adults in the United States: NHANES 2009 to 2012. *Journal of Periodontology*.

[7] Borgnakke W. S. (2015). Does treatment of periodontal disease influence systemic disease?. *Dental Clinics of North America*.

[8] Nadeem M., Stephen L., Schubert C., Davids M. R. (2009). Association between periodontitis and systemic inflammation in patients with end-stage renal disease. *SADJ*.

[9] Seymour G. J., Ford P. J., Cullinan M. P., Leishman S., Yamazaki K. (2007). Relationship between periodontal infections and systemic disease. *Clinical Microbiology and Infection*.

[10] Yamaguchi Y., Kurita-Ochiai T., Kobayashi R., Suzuki T., Ando T. (2015). Activation of the NLRP3 inflammasome in *Porphyromonas gingivalis*-accelerated atherosclerosis. *Pathogens and Disease*.

[11] Chukkapalli S. S., Rivera M. F., Velsko I. M. (2014). Invasion of oral and aortic tissues by oral spirochete *Treponema denticola* in ApoE(-/-) mice causally links periodontal disease and atherosclerosis. *Infection and Immunity*.

[12] Rivera M. F., Lee J.-Y., Aneja M. (2013). Polymicrobial infection with major periodontal pathogens induced periodontal disease and aortic atherosclerosis in hyperlipidemic ApoE(null) mice. *PLoS ONE*.

[13] Lee H.-R., Jun H.-K., Kim H.-D., Lee S.-H., Choi B.-K. (2012). *Fusobacterium nucleatum* GroEL induces risk factors of atherosclerosis in human microvascular endothelial cells and ApoE^−/−^ mice. *Molecular Oral Microbiology*.

[14] Daniel R., Gokulanathan S., Shanmugasundaram N., Lakshmigandhan M., Kavin T. (2012). Diabetes and periodontal disease. *Journal of Pharmacy & Bioallied Sciences*.

[15] Lakschevitz F., Aboodi G., Tenenbaum H., Glogauer M. (2011). Diabetes and periodontal diseases: interplay and links. *Current Diabetes Reviews*.

[16] Preshaw P. M., Bissett S. M. (2013). Periodontitis: oral complication of diabetes. *Endocrinology and Metabolism Clinics of North America*.

[17] Andriankaja O. M., Joshipura K. (2014). Potential association between prediabetic conditions and gingival and/or periodontal inflammation. *Journal of Diabetes Investigation*.

[18] Chang J. S., Tsai C., Chen L., Shan Y. (2016). Investigating the association between periodontal disease and risk of pancreatic cancer. *Pancreas*.

[19] Rosier B. T., De Jager M., Zaura E., Krom B. P. (2014). Historical and contemporary hypotheses on the development of oral diseases: are we there yet?. *Frontiers in Cellular and Infection Microbiology*.

[20] Hajishengallis E., Hajishengallis G. (2014). Neutrophil homeostasis and periodontal health in children and adults. *Journal of Dental Research*.

[21] Moutsopoulos N. M., Konkel J., Sarmadi M. (2014). Defective neutrophil recruitment in leukocyte adhesion deficiency type I disease causes local IL-17-driven inflammatory bone loss. *Science Translational Medicine*.

[22] Delima A. J., Van Dyke T. E. (2003). Origin and function of the cellular components in gingival crevice fluid. *Periodontology 2000*.

[23] von Vietinghoff S., Ley K. (2008). Homeostatic regulation of blood neutrophil counts. *Journal of Immunology*.

[24] Amulic B., Cazalet C., Hayes G. L., Metzler K. D., Zychlinsky A. (2012). Neutrophil function: from mechanisms to disease. *Annual Review of Immunology*.

[25] Summers C., Rankin S. M., Condliffe A. M., Singh N., Peters A. M., Chilvers E. R. (2010). Neutrophil kinetics in health and disease. *Trends in Immunology*.

[26] Weaver C. T., Elson C. O., Fouser L. A., Kolls J. K. (2013). The Th17 pathway and inflammatory diseases of the intestines, lungs, and skin. *Annual Review of Pathology*.

[27] Eskan M. A., Jotwani R., Abe T. (2012). The leukocyte integrin antagonist Del-1 inhibits IL-17-mediated inflammatory bone loss. *Nature Immunology*.

[28] Pelletier M., Maggi L., Micheletti A. (2010). Evidence for a cross-talk between human neutrophils and Th17 cells. *Blood*.

[29] Zenobia C., Hajishengallis G. (2015). Basic biology and role of interleukin-17 in immunity and inflammation. *Periodontology 2000*.

[30] Chavakis E., Choi E. Y., Chavakis T. (2009). Novel aspects in the regulation of the leukocyte adhesion cascade. *Thrombosis and Haemostasis*.

[31] Ley K., Laudanna C., Cybulsky M. I., Nourshargh S. (2007). Getting to the site of inflammation: the leukocyte adhesion cascade updated. *Nature Reviews Immunology*.

[32] Hajishengallis G., Chavakis T. (2013). Endogenous modulators of inflammatory cell recruitment. *Trends in Immunology*.

[33] Kolaczkowska E., Kubes P. (2013). Neutrophil recruitment and function in health and inflammation. *Nature Reviews Immunology*.

[34] Choi E. Y., Chavakis E., Czabanka M. A. (2008). Del-1, an endogenous leukocyte-endothelial adhesion inhibitor, limits inflammatory cell recruitment. *Science*.

[35] Martin C., Burdon P. C. E., Bridger G., Gutierrez-Ramos J.-C., Williams T. J., Rankin S. M. (2003). Chemokines acting via CXCR2 and CXCR4 control the release of neutrophils from the bone marrow and their return following senescence. *Immunity*.

[36] Stark M. A., Huo Y., Burcin T. L., Morris M. A., Olson T. S., Ley K. (2005). Phagocytosis of apoptotic neutrophils regulates granulopoiesis via IL-23 and IL-17. *Immunity*.

[37] Ryder M. I. (2010). Comparison of neutrophil functions in aggressive and chronic periodontitis. *Periodontology 2000*.

[38] Darveau R. P. (2010). Periodontitis: a polymicrobial disruption of host homeostasis. *Nature Reviews Microbiology*.

[39] Zenobia C., Luo X. L., Hashim A. (2013). Commensal bacteria-dependent select expression of CXCL2 contributes to periodontal tissue homeostasis. *Cellular Microbiology*.

[40] Deas D. E., Mackey S. A., McDonnell H. T. (2003). Systemic disease and periodontitis: manifestations of neutrophil dysfunction. *Periodontology 2000*.

[41] Hanna S., Etzioni A. (2012). Leukocyte adhesion deficiencies. *Annals of the New York Academy of Sciences*.

[42] de Haar S. F., Hiemstra P. S., van Steenbergen M. T. J. M., Everts V., Beertsen W. (2006). Role of polymorphonuclear leukocyte-derived serine proteinases in defense against *Actinobacillus actinomycetemcomitans*. *Infection and Immunity*.

[43] Eick S., Puklo M., Adamowicz K. (2014). Lack of cathelicidin processing in Papillon-Lefèvre syndrome patients reveals essential role of LL-37 in periodontal homeostasis. *Orphanet Journal of Rare Diseases*.

[44] Sørensen O. E., Clemmensen S. N., Dahl S. L. (2014). Papillon-Lefèvre syndrome patient reveals species-dependent requirements for neutrophil defenses. *The Journal of Clinical Investigation*.

[45] Nauseef W. M. (2014). Proteases, neutrophils, and periodontitis: the NET effect. *The Journal of Clinical Investigation*.

[46] Hart T. C., Atkinson J. C. (2007). Mendelian forms of periodontitis. *Periodontology 2000*.

[47] Harris E. S., Weyrich A. S., Zimmerman G. A. (2013). Lessons from rare maladies: leukocyte adhesion deficiency syndromes. *Current Opinion in Hematology*.

[48] Landzberg M., Doering H., Aboodi G. M., Tenenbaum H. C., Glogauer M. (2015). Quantifying oral inflammatory load: oral neutrophil counts in periodontal health and disease. *Journal of Periodontal Research*.

[49] Aboodi G. M., Goldberg M. B., Glogauer M. (2011). Refractory periodontitis population characterized by a hyperactive oral neutrophil phenotype. *Journal of Periodontology*.

[50] Chapple I. L. C., Matthews J. B. (2007). The role of reactive oxygen and antioxidant species in periodontal tissue destruction. *Periodontology 2000*.

[51] Scapini P., Cassatella M. A. (2014). Social networking of human neutrophils within the immune system. *Blood*.

[52] Wang J., Arase H. (2014). Regulation of immune responses by neutrophils. *Annals of the New York Academy of Sciences*.

[53] Dababneh R., Al-wahadneh A. M., Hamadneh S., Khouri A., Bissada N. F. (2008). Periodontal manifestation of leukocyte adhesion deficiency type I. *Journal of Periodontology*.

[54] Nussbaum G., Shapira L. (2011). How has neutrophil research improved our understanding of periodontal pathogenesis?. *Journal of Clinical Periodontology*.

[55] Nualart Grollmus Z. C., Morales Chávez M. C., Silvestre Donat F. J. (2007). Periodontal disease associated to systemic genetic disorders. *Medicina Oral, Patología Oral y Cirugía Bucal*.

[56] Sutton C. E., Lalor S. J., Sweeney C. M., Brereton C. F., Lavelle E. C., Mills K. H. G. (2009). Interleukin-1 and IL-23 induce innate IL-17 production from *γδ* T cells, amplifying Th17 responses and autoimmunity. *Immunity*.

[57] Sato K., Suematsu A., Okamoto K. (2006). Th17 functions as an osteoclastogenic helper T cell subset that links T cell activation and bone destruction. *The Journal of Experimental Medicine*.

[58] Koenders M. I., van den Berg W. B. (2015). Novel therapeutic targets in rheumatoid arthritis. *Trends in Pharmacological Sciences*.

[59] Hajishengallis G. (2014). The inflammophilic character of the periodontitis-associated microbiota. *Molecular Oral Microbiology*.

[60] Allam J.-P., Duan Y., Heinemann F. (2011). IL-23-producing CD68(+) macrophage-like cells predominate within an IL-17-polarized infiltrate in chronic periodontitis lesions. *Journal of Clinical Periodontology*.

[61] Hajishengallis G., Chavakis T., Hajishengallis E., Lambris J. D. (2015). Neutrophil homeostasis and inflammation: novel paradigms from studying periodontitis. *Journal of Leukocyte Biology*.

[62] Taylor P. R., Roy S., Leal S. M. (2014). Activation of neutrophils by autocrine IL-17A-IL-17RC interactions during fungal infection is regulated by IL-6, IL-23, ROR*γ*t and dectin-2. *Nature Immunology*.

[63] Yago T., Nanke Y., Ichikawa N. (2009). IL-17 induces osteoclastogenesis from human monocytes alone in the absence of osteoblasts, which is potently inhibited by anti-TNF-*α* antibody: a novel mechanism of osteoclastogenesis by IL-17. *Journal of Cellular Biochemistry*.

[64] Chakravarti A., Raquil M.-A., Tessier P., Poubelle P. E. (2009). Surface RANKL of Toll-like receptor 4-stimulated human neutrophils activates osteoclastic bone resorption. *Blood*.

[65] Shin J., Maekawa T., Abe T. (2015). DEL-1 restrains osteoclastogenesis and inhibits inflammatory bone loss in nonhuman primates. *Science Translational Medicine*.

[66] Lakschevitz F. S., Aboodi G. M., Glogauer M. (2013). Oral neutrophil transcriptome changes result in a pro-survival phenotype in periodontal diseases. *PLoS ONE*.

[67] Hajishengallis G., Lamont R. J. (2014). Breaking bad: manipulation of the host response by *Porphyromonas gingivalis*. *European Journal of Immunology*.

[68] Maekawa T., Krauss J. L., Abe T. (2014). *Porphyromonas gingivalis* manipulates complement and TLR signaling to uncouple bacterial clearance from inflammation and promote dysbiosis. *Cell Host and Microbe*.

[69] Burns E., Eliyahu T., Uematsu S., Akira S., Nussbaum G. (2010). TLR2-dependent inflammatory response to *Porphyromonas gingivalis* is MyD88 independent, whereas MyD88 is required to clear infection. *Journal of Immunology*.

[70] Liang S., Krauss J. L., Domon H. (2011). The C5a receptor impairs IL-12-dependent clearance of *Porphyromonas gingivalis* and is required for induction of periodontal bone loss. *The Journal of Immunology*.

[71] Popadiak K., Potempa J., Riesbeck K., Blom A. M. (2007). Biphasic effect of gingipains from *Porphyromonas gingivalis* on the human complement system. *The Journal of Immunology*.

[72] Jones S. A., Sutton C. E., Cua D., Mills K. H. G. (2012). Therapeutic potential of targeting IL-17. *Nature Immunology*.

[73] Hajishengallis G., Maekawa T., Abe T., Hajishengallis E., Lambris J. D. (2015). Complement involvement in periodontitis: molecular mechanisms and rational therapeutic approaches. *Advances in Experimental Medicine and Biology*.

[74] Maekawa T., Abe T., Hajishengallis E. (2014). Genetic and intervention studies implicating complement C3 as a major target for the treatment of periodontitis. *The Journal of Immunology*.

[75] Lalla E., Papapanou P. N. (2011). Diabetes mellitus and periodontitis: a tale of two common interrelated diseases. *Nature Reviews Endocrinology*.

